# Progression of HIV-1 CKDs: Viral Load Versus *APOL1* Risk Alleles

**DOI:** 10.1016/j.ekir.2025.01.005

**Published:** 2025-01-10

**Authors:** Patricio E. Ray

**Affiliations:** 1 Child Health Research Center and Department of Pediatrics, University of Virginia School of Medicine, Charlottesville, Virginia, USA

See Clinical Research on Page 855

People living with HIV-1 of sub-Saharan West African ancestry develop chronic kidney diseases (CKDs) more frequently than other groups with or without HIV-1 infection.^1^^,^^2^ A large fraction of this disparity is explained by 2 coding sequence variants in the *APOL1* gene known as G1 and G2, which are associated with focal segmental glomerulosclerosis, HIV-associated nephropathy (HIVAN), and hypertension attributed end-stage kidney diseases.^3^ How the *APOL1* risk alleles (RAs) affect the development of hypertension, cardiovascular diseases (CVDs), and CKD only in people living with HIV-1 on antiretroviral therapies (ART) is unclear at the present time. In this issue of the journal, Tassiopoulos *et al.* explored the associations between the *APOL1* RAs and hypertension, CVD and CKD in Black adult persons with HIV (*n* = 1194) who were followed-up for up to 20 years.^4^ As expected, they found that their estimated glomerular filtration rate (eGFR) declined more significantly in people carrying 2 copies of the *APOL1* RAs (G1/G1, G2/G2, or G1/G2) and was greater in those who did not achieve sustained viral suppression. A similar association was noted in those carrying only 1 copy of the *APOL1* RAs during the second decade of follow-up. In contrast, no associations between the *APOL1* RAs and hypertension or CVD incidents were found.

A significant contribution of Tassiopoulos *et al.* was their exclusive focus on Black adults living with HIV on ART and the finding that those carrying only 1 copy of the RAs were at higher long-term risk of developing CKD.^4^ Their findings support the results of a previous study that showed that the odds ratios for developing HIVAN in people carrying 2 copies of the *APOL1* RAs were 29 (95% confidence interval:13–68), whereas carrying 1 G1 allele showed a marginal significant association (odds ratio: 1.9, 95% confidence interval:1.01–3.5), and those with a single copy of the G2 allele showed no significant association.^5^ The latter HIVAN study however, included people followed-up during the early years of the AIDS epidemic who did not benefit from the new ART; therefore, the frequency and severity of HIV-CKD in this group were much higher.^5^ As expected, Tassiopoulos *et al.* showed that the renal effects of the *APOL1* RAs were modulated by the viral load.^4^ However, sustained viral suppression (defined by an HIV-RNA < 200 copies/ml for > 90% of follow-up time) reduced, but did not eliminate the association of the RAs with the eGFR decline. These results are in agreement with the notion that a high viral load is the major factor affecting the progression of HIV-CKD. Nonetheless, whether a high viral load plays such role by activating the innate immune response leading to the release of INF-γ and TNF-α that increase the expression and cytotoxicity of *APOL1* in podocytes and renal endothelial cells, or by facilitating the infection of renal epithelial cells, or both processes,^6^ are still open questions that warrant further investigation. Finally, their findings strongly suggest that the *APOL1-G0* genotype did not provide a protective effect against HIV-CKD, given that the estimated GFR declined in people carrying 1 RA, further supporting the notion that the *APOL1* RAs are gain-of-function mutations.

There are several caveats that are worth taken into consideration when interpreting the study of Tassiopoulos *et al.* First, proteinuria was not assessed in all participants; thus, the diagnosis of CKD was based on estimated GFR criteria only. It is worth mentioning that most people in the sustained viral suppression group showed mean estimated GFR levels above the CKD threshold of 60 ml/min per 1.73 m^2^ regardless of the number of *APOL1* RAs. Furthermore, the association of the *APOL1* RAs with proteinuria was eliminated in the sustained viral suppression group, whereas participants carrying the *APOL1* RAs in the nonsustained viral suppression groups showed more severe proteinuria. Taking together, these findings confirm that the viral load is the critical factor determining the progression of HIV-CKD (Figure 1). It is worth mentioning here that people of sub-Saharan West African ancestry carrying 2 G0 alleles can develop HIVAN, albeit less frequently.^5^ Thus, other factors associated with sub-Sharan West African ancestry should play additional roles in HIVAN. Second, people who developed hypertension during the study period (31%, *n* = 267) had other risk factors for hypertension, including older age, higher body mass indexes, diabetes, and/or having received tenofovir disoproxil fumarate as a part of their initial ART regimen, making it more difficult to define the specific role of *APOL1*. Alternatively, the lack of association between the *APOL1* RAs and hypertension suggests that either the GFR decline was not enough to affect the blood pressure, or that some participants were undergoing the early stages of HIVAN. People undergoing the earlier stages of HIVAN do not typically develop hypertension despite multiple risk factors to do so.This finding can be explained, at least partially, by the tubulointerstitial lesions of HIVAN, which induce salt wasting states in humans and HIV-Tg mice.^6^^,^^8^ Early HIVAN and salt-losing tubulopathies are difficult to detect if proteinuria and other urinary losses are not monitored on a regular basis. Third, the number of people who developed a CVD incident was too small to reach definitive conclusions (2.7%, *n* = 31) and these people were older by a median of 12 years as well as more likely to have hypertension or a family history of CVD.

**Figure 1:**
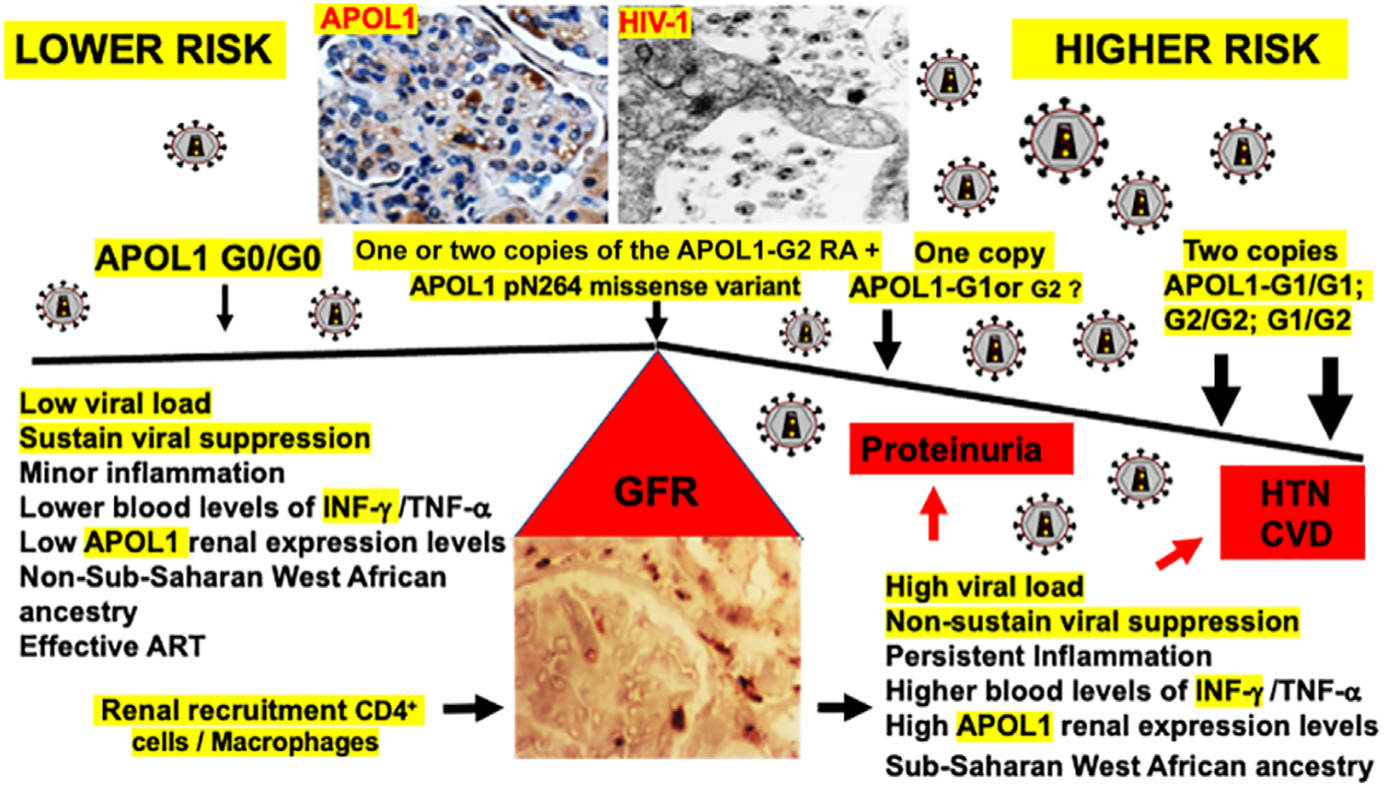
HIV-1 and the *APOL1* risk alleles interact to precipitate the progression of HIV-CKD in Black adults on ART. Carriers of 2 copies of the *APOL1* risk alleles with higher viral loads showed more significant proteinuria and estimated GFR declines and may have the highest long-term risk for developing hypertension and/or CVD in association with the progression of HIV-CKD. Carriers of a single copy of the G1 risk allele with higher viral loads showed more significant GFR declines when compared with those with sustained viral suppression. It remains unclear whether carriers of a single copy of the G2 risk allele will show similar outcomes than carriers of 1 copy of the G1 risk allele. People coinheriting the *APOL1* p.N264 missense variant with either 2 or 1 copy of the G2 allele may have a similar risk of CKD than those carrying 2 copies of the *APOL1-G0* alleles.^7^ ART, antiretroviral therapy; CKD, chronic kidney disease; CVD, cardiovascular disease; GFR, glomerular filtration rate; HTN, hypertension.

It is worth discussing here that the criteria used to define sustained viral suppression included participants who had persistent low-level viremia, defined by the World Health Organization as an HIV-RNA viral load between 51 and 999 HIV RNA copies/ml.^9^ A suppressed viral load, defined as an HIV-RNA viral load < 50 copies/ml, means that the virus is not replicating at detectable levels and therefore is considered the hallmark of successful treatments.^9^ Conversely, the virus continues to replicate in people with persistent low-level viremia, and these persons are more likely to develop virological failure, immune activation, microbial translocation, systemic inflammation, and have worse clinical outcomes relative to those with a suppressed viral load.^9^ A persistent low-level viremia indicates that people may have poor adherence, drug resistance, or hyperactive viral reservoirs.^9^ The latter can be identified using molecular virology tests to detect viruses undergoing clonal expansion. All these issues should be considered when following people living with HIV-1. Further studies are needed to define what viral load suppression levels are needed to prevent CKD in carriers of the *APOL1* RAs relative to those at lesser risk.

Finally, recent studies showed that the *APOL1* p.N264 missense variant, when coinherited with the G2 *APOL1* RA, significantly reduced the penetrance of the G1/G2 and G2/G2 RAs by converting them to a low-risk genotype^7^ (Figure 1). This protective effect, which was not seen with the G1 or G1/G1 risk genotype, may explain the lack of association between the G2 RA and HIVAN discussed earlier. Thus, it remains to be determined whether people living with HIV-1 carrying 1 copy of the G2 allele are at a similar risk of GFR decline compared to those carrying 1copy of the G1 allele. Notably, *APOL1-G1* is the RA more frequently associated with HIVAN.^2^ In conclusion, Tassiopoulos *et al.* showed that the *APOL1* RAs play a role in enhancing the progression of HIV-CKD, underscoring the importance of suppressing the viral load to ameliorate this process in Black adults on ART.^4^ Their study will hopefully stimulate further research to define the best way of accomplishing this goal.

## Disclosure

The author declared no competing interests.
